# Designing a customized clinical practice guideline regarding antibiotic prophylaxis for Iranian general dentists

**DOI:** 10.1186/s12903-019-0905-3

**Published:** 2019-10-07

**Authors:** Najmeh Savadi, Omid Barati, Hossein Mirhadi, Ali Golkari

**Affiliations:** 1 Department of Dental Public Health, School of Dentistry, Shiraz University of Medical Sciences, Ghasrdasht Street, Shiraz, 71956-15878 Iran; 2 Education and Development Center, Health Human Resources Research Center, Iran University of Medical Sciences, Tehran, Iran; 3 Department of Endodontics, School of Dentistry, Shiraz University of Medical Sciences, Shiraz, Iran

**Keywords:** Antibiotic prophylaxis, Clinical practice guideline, Dental care, Infective endocarditis, Prosthetic joint

## Abstract

**Background:**

Clinical practice guidelines produced by developed countries seemed to be not completely feasible for developing countries due to their different local context. In this study, we designed a customized guideline about antibiotic prophylaxis before dental procedures for Iranian general dentists.

**Methods:**

This study was conducted of two parts, including a qualitative part and a cross-sectional analytic part. A multidisciplinary team searched for related guidelines and other documents, selected the most updated and high quality ones, customized their recommendations based on available antibiotics in Iran, prepared a draft adapted guideline and summarized its recommendations in 3 flowcharts. An expert panel (20 specialists of four Iranian dental universities) participated in a consensus process, afterwards to determine the relevance and clarity of the flowcharts and their items. Then the Content Validity Indices (CVIs) were calculated and any items with CVI higher than 0.79 remained.

**Results:**

The adapted recommendations were summarized in flowcharts A to C. Two separate groups of patients who need antibiotic prophylaxis were presented in flowchart A; including those with high risk for distant-site infection (infective endocarditis and prosthetic joint infection) and those at risk for poor healing and orofacial infection (due to impaired immunologic function). Flowcharts B and C described antibiotic regimen and also the dental procedures where antibiotic prophylaxis was needed for mentioned groups. The content validity indices and the percentages of agreement between the expert panel members were considerably high.

**Conclusions:**

A localized, clear and straight forward guideline that addresses all groups of dental patients who need antibiotic prophylaxis has been produced for Iranian general dentists.

**Supplementary information:**

**Supplementary information** accompanies this paper at 10.1186/s12903-019-0905-3.

## Background

Deciding about the best prescription for patients might be confusing. Clinical practice guidelines (CPGs) provide evidence based recommendations to help practitioners select the best treatment plan and the most effective drug regimen. Some international organizations and developed countries have already prepared and asserted several CPGs [1, 2]. However, different prescription patterns may be needed in other countries/communities due to drug resistance, social, cultural, economic, and/or promotional factors [3, 4]. Availability, accessibility and affordability of prescribed drugs are also other factors [5, 6] which prevent using the same practice guideline recommendations in different countries; thus designing a localized CPG seems critical for each country.

Antibiotics are one of the most common medications prescribed by dental practitioners [7]. They are commonly prescribed for therapeutic purposes to manage oral infections [8–12]. Sometimes prescriptions are given out or recommended as prophylaxis for the prevention of distant-site infections before dental procedures [9, 13, 14]. Infective Endocarditis (IE) [15] and prosthetic joint infection (PJI) [16] are examples of medical conditions which are concerned that might occur in susceptible patients following dental treatment. The scientific basis for prophylaxis was to eliminate or reduce transient bacteremia caused by invasive dental procedures [17]. Patients with a compromised immune system are in the center of this kind of concerns as they may be less able to tolerate a transient bacteremia and are prone to orofacial infection following invasive dental procedures [18]. Some health scientists insist that special considerations should be paid to patients with these medical conditions. And therefore, it is important to implement strategies to prevent development of infections during dental treatments in these patients [19, 20].

Antibiotic prophylaxis (AP) has been considered to be safe and cost-effective. Dental practitioners have welcomed antibiotic prophylaxis regimen partly due to the fear of being blamed. However, they have also been confused about the correct prescription at the same time, as they do not deal with such patients as a routine manner.

The main potential problems of widely using AP are risk of adverse drug reactions, costs for the health-care system and risk of promoting antibiotic resistance [15, 21]. Although the cost of antibiotic prophylaxis for an individual might be low, the potential cost for the health-care system of a country is considered high, even millions of dollar per year [21]. Furthermore, one of the biggest threats to human health in today’s world is resistance to the antibiotics, according to the world health organization [22, 23]. Antibiotic resistance is defined as the ability of bacteria to change and resist against the effects of antibacterial agent [24, 25]. The loss of antibiotics’ efficacy against bacteria could lead towards consumption of more expensive antibiotics in high-income countries. However, in low and middle-income countries, antibiotic resistance might increase morbidity and mortality because affordability of second-line drugs limits their use [25].

Variations in antibiotic resistance between countries can be attributed to different volumes and patterns of antibiotic consumption, whether this use is appropriate or not [26, 27]. Due to uncertainty of medical laws and unawareness of updated guidelines, general dentists are usually confused about the indication of AP, correct dose and time of administration. They often trust to cautious practitioners’ recommendations who suggest prophylaxis in any doubtful disappointing situations based on their own individual experiences [28]. Either inadequate knowledge or poor compliance with the available guidelines has been reported in many studies [29–31]. Despite widely available CPGs about AP, dentists are not always aware of the most current ones [32]. Studies have revealed that Iranian dentists have moderate level of knowledge regarding the current AP guidelines. But, they use CPGs related to other countries’ organizations by their personal choice [33–35].

Therefore, there is a need to a readily accessible, problem-orientated CPG which should be matched with each community population’s needs. Therefore, the aim of this study is to design and adapt a guideline about AP, giving Iranian general dentists the recommendations on when, which and in what dosage the antibiotics should be prescribed.

## Methods

This study consisted of a qualitative part and a cross-sectional analytic part. It was carried out during the three main phases (Set-up phase, Adaptation phase and Finalization phase) [36] by a multidisciplinary team to oversee the adaptation process. In the first phase, a multidisciplinary team including a dental public health specialist, an oral medicine specialist, an endodontist, a periodontist, a general dentist, a pharmacologist, a PhD of biostatistics and an expert searcher were selected by convenience sampling and familiarized with the study. This team was served as a focus group in the qualitative phase of the study. The Adaptation phase was started through the process of searching and retrieving guidelines about AP in dentistry, assessing the guideline quality, decision making about adaptation, and preparing the draft adapted guideline. The interviews and meetings were conducted by a female dental public health PhD candidate who was trained and calibrated for this kind of qualitative data collection in advance. She had no relationship with any of the invited participants.

### Search for guidelines and other relevant documents

Two themes were considered in advance for searching CPGs and other documents. One theme investigated AP in different group of patients including those with cardiac conditions, with prosthetic joints and with impaired immunologic functions. The other theme considered AP for adults and children, separately. Since CPGs might not be published in journals; we started the search for guidelines about AP since 2005 in guideline clearinghouses such as the National Institute for Clinical Excellence (NICE), the American Dental Association (ADA), the Royal College of Surgeons, the Scottish Dental Clinical Effectiveness Program, etc.

An additional search in a broader database such as MEDLINE, Springer, Elsevier and Cochran Library using a standardized search strategy was done for more guidelines. Following terms were used: practice guideline [Publication Type] OR guideline [Title], in combination with words related to antibiotic prophylaxis [Title] AND dental care OR dentistry OR Infective Endocarditis OR Prosthetic joint OR immunosuppression OR immunocompromised patients. Internet search engines such as Google and Yahoo were also used to find guidelines. A recent study indicated that available CPGs on the Internet had equal or higher quality than those published in the periodical literature [37]. A further search was also done to identify other recent and relevant documents such as recent meta-analysis or systematic reviews. These documents might be used to supplement the recommendations of retrieved guidelines.

### Selection between guidelines and recommendations to generate an adapted guideline

The following characteristics of the retrieved CPGs were summarized in a table: developing organization/authors, date of publication, country/language of publication and the practice guideline’s recommendations. Those CPGs which were not in English language, or were based on approvals made before 2005, and those which were outdated by a later update of the same organization were excluded.

### Preparation of a draft of the adapted guideline

The focus group was asked to discuss and write their opinions about selected guidelines and recommendations. The meeting was continued until a clear conclusion was obtained. The final recommendations were attained using content analysis and coded by one calibrated coder. All transcripts returned to multidisciplinary team for comment and/or correction. The recommendations were subsequently localized based on available antibiotic forms in Iran. Antibiotics’ forms and dosage were checked by pharmacologists of Food and Drug Department of Shiraz medical university. The draft of all customized recommendations and related data were prepared and summarized in the Flowcharts A to C (Additional files 1, 2, and 3). Then, all flowcharts were examined by the selected multidisciplinary team and were revised for the second time to gain the best relevant, simple and clear recommendations for general dentists.

### Consensus process (external peer review)

Each dentist member of the multidisciplinary team (five people) was asked to recommend five dental specialists among Iranian university professors. Due to three duplicate names among the twenty-five introduced professors, twenty-two ones were invited for expert panel. All invited participants were informed about the study’s aim, scope and process, and the interests of the interviewer, by written materials printed and sent to them. Verbal discussion over phone or in person was followed when necessary. Since two of the specialists refused to participate in the consensus process, the study was continued with twenty ones. The selected dental specialists including 2 dental public health specialists, 5 oral medicine specialists, 3 oral and maxillofacial surgeons, 3 periodontists, 3 endodontists, 1 pediatric dentist, 1 orthodontist, 1 prosthodontist and 1 restorative specialist were from four Iran universities (Tehran, Shiraz, Yazd and Bushehr University of Medical Sciences). Written informed consent was obtained from each participant in advance to their participation. All meetings were conducted in dental schools.

Three questionnaires were designed to assess the relevance and clarity of different items of flowcharts. The questionnaires consisted of 6, 12 and 14 questions for flowcharts A, B and C, respectively. A 4-point ordinal scale was considered for each question to avoid a neutral midpoint [38]. Labels and the item-rating range for the four points were very relevant = 4, relevant but need minor revision = 3, the item need some revision = 2, not relevant =1 for examining relevance and very clear = 4, clear but need minor revision = 3, the item need some revision = 2, not clear = 1 for examining clarity of each item [39].

The expert panel was asked to determine the relevance and clarity of each item by selecting one of a 4 point ordinal scale and to write any revision if needed based on their knowledge and experience. The Content Validity Index (CVI) [38, 40–42] was calculated for each item and for the flowchart as a whole, afterwards. For the Item-level-CVI (I-CVI), values range from 0 to 1. whenever I-CVI > 0.79, the item was considered relevant, between 0.70 and 0.79, the item might need some revisions, and if the value was below 0.70 the item was deleted [38, 42]. The Scale-level-CVI (S-CVI) was computed by two methods [42], one was the Universal Agreement (UA) among experts (S-CVI/UA), and the second, the Average CVI (S-CVI/Ave) [42]. A criterion of 0.80 was often used as the lower limit of acceptability for an S-CVI [40, 43]. The UA method was calculated by adding all I-CVI’s equal to 1.00 divided by number of items; while the Average approach averaged the I-CVIs by summing them and dividing by the number of items.

### Production of the final guidance document

In the last phase, all flowcharts were re-evaluated; any items with CVI higher than 0.79 were remained [38] and any needed revision were done. The multidisciplinary team concluded that enough data were gathered and there was no need for invitation of new participants. This last version of flowcharts presented as final guideline product for Iranian general dentists.

## Results

Twenty two guidelines and systematic reviews were obtained as search results. Five guidelines were excluded and seventeen most recently updated guidelines and systematic reviews which were fully available, had structural soundness and met our inclusion/exclusion criteria were selected and used in this study. The results of adapted recommendations were summarized in Flowcharts A to C (Additional files 1, 2, and 3). Flowchart A described two distinct groups of patients who need AP. That included those with high risk for systemic/distant-site infection (IE and PJI) and those at risk for poor healing and orofacial infection (due to impaired immunologic function). Flowcharts B and C presented antibiotic regimen for groups in need of AP (described in Flowchart A) and also the dental procedures for which AP should be recommended.

Two main groups of patients who need AP were:

### I-patients at risk for systemic/distant-site infection

In the recent years, the concept of AP to prevent these infections has changed considerably. Reviewing relevant literature indicated that not only antibiotic regimen for prophylaxis has become simpler and shorter, but also the number of procedures and individuals for whom AP was recommended has significantly reduced.

### I-I- patients with specific cardiac conditions associated with the highest risk of IE

A 2013 Cochrane systematic review found weak evidences about the effectiveness of AP against IE in persons at risk who experienced an invasive dental procedure [44]. Due to lack of evidence either for or against AP and the extensive risks of antibiotic resistance and adverse reaction (anaphylaxis), guideline committees like the European Society of Cardiology [45] and the AHA [46] concluded that it was safer to restrict AP to those patients seemed to be at the highest risk (Flowchart A-Box 1). In the UK, however, AP was forbidden for all invasive procedures in 2008 according to recommendations of the NICE guideline [14, 47]. A significant increase in cases of IE subsequently led to a subtly change in the guidance [48] which stated that “AP against IE is not recommended routinely for people undergoing dental procedures” [49]. This minor change indicated that in individual cases, AP might be appropriate; but it didn’t specify the antibiotic regimen, the individuals and the dental procedures for AP [15, 48, 50]. Yet, findings of a recent study which was reported cost-effectiveness of AP especially for those at high risk clearly supported the guidelines recommending AP for high-risk individuals [51].

### I-II- patients with prosthetic joints at potential increased risk of PGI

The 2014 Panel of the ADA and the AAOS proposed that in general, AP are not recommended before dental treatment for patients with prosthetic joints (PJs) due to lack of evidence for association between dental procedures and the occurrence of PJI [16]. However, they added certain exceptions to be considered in decision making for AP [52]. These exceptions included some systemic comorbid conditions that might prone patients with PJs to infection following bacteremia caused by invasive dental procedures (Flowchart A-Box 1). In these situations, yet, the mentioned guideline committees recommended that general dentists consult with the patient’s physician to make better judgment for AP in patients with PJs [16].

### II- patients at risk for poor healing and orofacial infection due to impaired immunologic function

There are many medical conditions associated with suppression of the immune system including some underlying diseases and medication for managing these diseases [53]. Since several documents reported the mouth as the most common source of infection and sepsis in immunosuppressed patients; AP appeared appropriate for these individuals before invasive dental procedures [18]. The National Cancer Institute suggested that AHA regimen for AP could also be prescribed for immunosuppressed patients with Absolute neutrophil count (ANC) < 1000 [54]. The ANC is an important marker for quantifying a patient’s severity of immunosuppression [53]. However, these recommendations are more based on medico-legal concerns and there is still weak scientific evidence for supporting the practice of routine AP before invasive dental procedures in immunocompromised patients [53].

The results of content validity tests of the mentioned flowcharts are presented in Tables 1, 2, 3. In addition to the I-CVI, the S-CVI/UA and S-CVI/Ave indices are reported in the tables. Table 1 indicates that Flowchart A and its items were relevant and clear. All I-CVIs were larger than the threshold value (> 0.79) ranging from 0.95 to 1.00. Moreover, the S-CVI/Ave (0.9917) and S-CVI/UA (0.8333) were remarkably large.

**Table 1:** Content validity indices for part A of the flowchart

	Items	I-CVI	
		Relevance	Clarity
1	Assess patient’s medical history	1.00	1.00
2	Is the patient high risk for distant-site infection? (Refer to Box 1)	1.00	1.00
3	Box 1	1.00	1.00
4	Is the patient at risk for poor healing and systemic spread of orofacial infection to impaired immunologic function? (Refer to Box 2)	1.00	1.00
5	Box 2	0.95	0.95
6	No need for antibiotic prophylaxis Do any required dental treatment	1.00	1.00
	S-CVI/Ave	0.9917	0.9917
	S-CVI/UA	0.8333	0.8333

*I-CVI* Item-level Content Validity Index, *S-CVI/Ave* Scale-level Content Validity Index, Averaging calculation method, *S-CVI/UA* Scale-level Content Validity Index, Universal Agreement calculation method

**Table 2:** Content validity indices for part B of the flowchart

	Items	I-CVI	
		Relevance	Clarity
1	Does patient need invasive dental procedures? (Refer to Box 1)	0.95	1.00
2	Box 1	1.00	1.00
3	No need for antibiotic prophylaxis Do any required dental treatment	1.00	1.00
4	Need for multiple dose antibiotic prophylaxis: A single high dose 30 to 60 min before dental procedure, following with usual dose for 5–7 days (See also Box 2)	1.00	1.00
5	Does patient have history of allergy to Penicillin?	0.95	1.00
6	Is patient able to take oral medication?	1.00	1.00
7	Amoxicillin 2 g orally (4 × 500 mg Cap) (For children, refer to Box 4)	1.00	1.00
8	Ampicillin 2 g IM or IV (2 × 1 g vial) OR Cefazolin or Ceftriaxone 1 g IM or IV (For children, refer to Box 4)	1.00	1.00
9	Cephalexin 2 g orally (4x500mg Cap) OR Clindamycin 600 mg orally (2x300mg Cap) OR Azithromycin 500 mg orally OR Clarithromycin 500 mg orally (For children, refer to Box 4)	1.00	1.00
10	Clindamycin 600 mg IM or IV (For children, refer to Box 4)	1.00	1.00
11	Box 2	1.00	1.00
12	Box 3	1.00	1.00
	S-CVI/Ave	0.9917	1.00
	S-CVI/UA	0.8333	1.00

*I-CVI* Item-level Content Validity Index, *S-CVI/Ave* Scale-level Content Validity Index, Averaging calculation method, *S-CVI/UA* Scale-level Content Validity Index, Universal Agreement calculation method

**Table 3:** Content validity indices for part C of the flowchart

	Items	I-CVI	
		Relevance	Clarity
1	Does patient need invasive dental procedures? (Refer to Box 1)	1.00	1.00
2	Box 1	1.00	1.00
3	No need for antibiotic prophylaxis Do any required dental treatment	1.00	1.00
4	Need for multiple dose antibiotic prophylaxis: A single high dose 30 to 60 min before dental procedure, following with usual dose for 5–7 days (See also Box 2 and 3)	1.00	1.00
5	Does patient have history of allergy to Penicillin?	1.00	1.00
6	Is patient able to take oral medication?	1.00	1.00
7	Amoxicillin 2 g orally (4 × 500 mg Cap) (For children, refer to Box 4)	1.00	1.00
8	Ampicillin 2 g IM or IV (2 × 1 g vial) OR Cefazolin or Ceftriaxone 1 g IM or IV (For children, refer to Box 4)	1.00	1.00
9	Cephalexin 2 g orally (4x500mg Cap)OR Clindamycin 600 mg orally (2x300mg Cap) OR Azithromycin 500 mg orally OR Clarithromycin 500 mg orally (For children, refer to Box 4)	1.00	1.00
10	Clindamycin 600 mg IM or IV (For children, refer to Box 4)	1.00	1.00
11	After any required invasive dental procedures: Amoxicillin Cap 500 mg, q8hOR Clindamycin 300 mg, q6h (for allergic patients)		
12	Box 2	1.00	1.00
13	Box 3	1.00	1.00
14	Box 4	1.00	1.00
	S-CVI/Ave	1.00	1.00
	S-CVI/UA	1.00	1.00

*I-CVI* Item-level Content Validity Index, *S-CVI/Ave* Scale-level Content Validity Index, Averaging calculation method, *S-CVI/UA* Scale-level Content Validity Index, Universal Agreement calculation method

For the 12-item Flowchart B, 99 and 100% of experts determined items as relevant and clear, respectively. About 83% of items achieved relevance rating of 3 or 4 by all experts; while for the clarity, the universal agreement of 100% was obtained. Table 3 displayed excellent relevance and clarity for the Flowchart C items. All I-CVIs were 1.00. Likewise, 100% agreement was found between all experts regarding the overall flowchart’ relevance and clarity. All items of the three flowcharts had acceptable CVI values of higher than 0.79. Therefore, none of the items were eliminated. Minor revisions were made in flowchart sentences. The last versions of flowcharts which are presented here as Additional files 1, 2, and 3 were considered as final guideline products for Iranian general dentists.

## Discussion

The present study customized the recommendations of updated CPGs regarding AP for Iranian general dentists. Several guidelines including those from the American Heart Association (AHA) [46], the American Academy of Orthopaedic Surgeons (AAOS) [16], the ADA [16] and the American Academy of Pediatric Dentistry (AAPD) [18, 54] were carefully studied and used as reference guidelines. The adopted recommendations were converted to simple and practical flowcharts. The reports from the Iranian expert panel, who evaluated relevance and clarity of the flowcharts, revealed high levels of content validity. The percentages of agreement between the expert panel members were also surprisingly high.

Although other guidelines about AP existed for specific groups, the flowchart presented in the current study would be one of the few guidelines which presented all groups who need AP together. Any dentist, a general practitioner or specialist, can simply use these flowcharts. Using Flowchart A, the two distinct groups who need AP can easily be distinguished. The dentist can easily move to Flowchart B or C, afterwards and follow the instructions for prescribing the AP.

The present study suggested that the antibiotic regimens supported by the AHA for cardiac indications might be appropriate for preventing PJI (Flowchart B) in all susceptible patients. The underlying mechanism of bacteria spreading from mouth to prosthetic joints (PJ) was the same as IE, though the anatomy, microbiology and pathogenesis of PJI and IE were different [52]. Furthermore, when dental procedures were performed for immunocompromised patients (Flowchart C), multiple-dose AP involving routine single dose AP plus postoperative antibiotic regimen during the week of healing might be necessary [52–54]. In recent years, several studies reported an increased potential for developing IE [55, 56] and PJI [57, 58] in risky patients with poor oral hygiene following routine activities like toothbrushing. Thus, good oral hygiene and regular dental visits should be considered as part of the prophylactic regimen and as important as AP.

Recommended prophylactic antibiotic regimens in this study are all based on nationally available forms of antibiotics in Iran. For Amoxicillin for example, four 500 mg capsules are stated in the flowcharts as this is the most available form of this antibiotic. This is while in some countries like the UK, there are ready-to-use sachets of Amoxicillin powder [48]. Furthermore, the antibiotic regimen for children based on their weight was reported in a separate box in both flowcharts.

Prophylaxis was considered reasonable for both groups of patients mentioned in Flowchart A before invasive dental procedures which manipulated the gingival tissue or the periapical region of teeth or perforated the oral mucosa [16, 18, 46, 50, 52]. However, in some immunocompromised patients, elective dental therapies should not be offered during severe immunosuppression phase. Furthermore, in dental emergency situations, considering the individual patient’s conditions and consulting with his/her physician for required supportive medical therapies like AP would be helpful [54]. Undoubtedly, AP especially for immunocompromised patients was driven by a combination of factors including the available CPGs, long-standing belief and practice habits of dentists and physicians and also medicolegal considerations [53].

The present study introduced a national and adapted guideline regarding AP for Iranian general dental practitioners. The introduction of antibiotic prescribing guidelines in England could improve general dentists’ practice in antibiotic administration and reduce the number of inappropriate prescriptions [59]. Expressing the present guidelines’ recommendations in forms of flowcharts could help general dentists to get the information better, easier and faster. Introducing this national CPG and re-auditing after a few years could be an initial step in implementing rational antibiotic use in Iran. Therefore, it is recommended for future studies to hold an educational program using this adapted guideline and to evaluate the feedback afterwards in order to reduce inappropriate and inaccurate antibiotic prescribing among Iranian general dental practitioners.

To move towards evidence-based practice in developing countries, it is better to emphasis on guideline adaptation and use rather than guideline development. Although guideline adaptation seemed simpler than guideline development, it was a complex time-consuming process (12 to even 24 months) needing more resources, support and facilitation than expected. Advanced methodological skills would be required to find and to appraise high quality documents. Furthermore, one favorable feature of the present guideline was diverse composition of the multidisciplinary team and the expert panel. Although this diversity led to more difficulties in arrangements, it played an important role to participate all involved specialists of dental universities’ departments in the process of guideline adaptation. The recommendations of this guideline were based on high levels of scientific evidence as well as complete consensus of the expert panel. Therefore, these recommendations, presented as flowcharts, could be confidently recommended for Iranian general dentists. It is important to remember that guideline adaptation was not an episodic activity and planning for periodically updating should be considered in all communities.

## Conclusion

In the present study the recommendations retrieved from updated CPGs regarding AP, were adopted and were converted to simple flowcharts for easier and faster access of Iranian general dentists. The flowcharts contained information about two distinct groups of patients who need AP, the antibiotic regimen for both adults and children and the dental procedures for which AP should be recommended. Relevance and clarity of the overall flowcharts and their items were evaluated by an Iranian expert panel. High content validity for the flowcharts was obtained and high percentages of agreement between the experts were reported.

## Additional files

**Additional file 1:** Our recommended flowchart for antibiotic prophylaxis for Iranian general dentists based on available antibiotics in Iran- Part A. (PDF 250 kb)

**Additional file 2:** Our recommended flowchart for antibiotic prophylaxis for Iranian general dentists based on available antibiotics in Iran Part B: patients at high risk for distant-site infection. (PDF 355 kb)

**Additional file 3:** Our recommended flowchart for antibiotic prophylaxis for Iranian general dentists based on available antibiotics in Iran Part C: patients at risk for poor healing and orofacial infection (Due to impaired immunologic function). (PDF 357 kb)

## Data Availability

The data used during the current study are available from the corresponding author on reasonable request.

## Abbreviations

AAOS: American Academy of Orthopaedic Surgeons
AAPD: American Academy of Pediatric Dentistry
ADA: American dental association
AHA: American Heart Association
ANC: Absolute neutrophil count
AP: Antibiotic prophylaxis
CPG: Clinical practice guideline
CVI: Content Validity Index
I-CVI: Item-level-CVI
IE: Infective endocarditis
NICE: National Institute for Clinical Excellence
PJ: Prosthetic joint
PJI: Prosthetic joint infection
S-CVI: Scale-level-CVI
S-CVI/Ave: Average CVI
S-CVI/UA: Scale-level-CVI/Universal Agreement
