# Supplementary material for: Designing a customized clinical practice guideline regarding antibiotic prophylaxis for Iranian general dentists
Source: BMC Oral Health. 2019 Oct 7;19:217. doi: 10.1186/s12903-019-0905-3 (PMC6781348; doi:10.1186/s12903-019-0905-3)
Supplement: Supplementary file 1 — Additional file 1: Our recommended flowchart for antibiotic prophylaxis for Iranian general dentists based on available antibiotics in Iran- Part A. (PDF 250 kb) [file 12903_2019_905_MOESM1_ESM.pdf]

Our recommended flowchart for antibiotic prophylaxis for Iranian general dentists based on available dose of antibiotics in Iran- Part A:

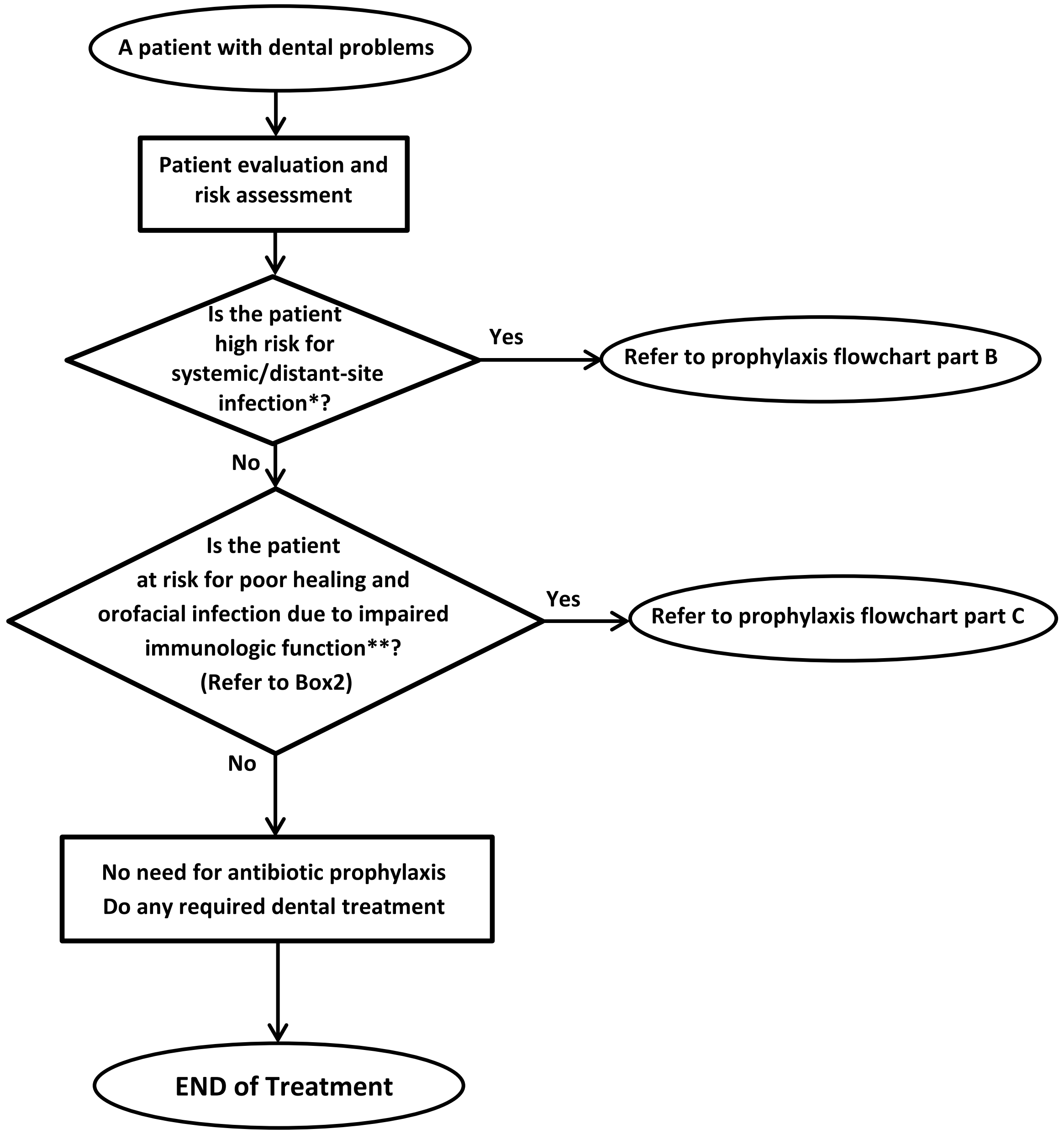

***Patients at risk for distant-site infection:**

1- Patients with specific cardiac conditions associated with the highest risk of Infective Endocarditis:

●Prosthetic cardiac valves, including trans-catheter implanted prostheses and homograft

●Prosthetic material used for cardiac valve repair, such as annuloplasty rings and chords

●Previous IE

●Unrepaired cyanotic congenital heart disease or repaired congenital heart disease, with residual shunts or valvular regurgitation at the site of or adjacent to the site of a prosthetic patch or prosthetic device.

●Cardiac transplant with valve regurgitation due to a structurally abnormal valve

2-Patients with prosthetic joints at potential increased risk of Prosthetic joint infection:

●Immunocompromised or immunosuppressed patients including:

- ✓ inflammatory arthropathies such as rheumatoid arthritis, systemic lupus erythematosus
- ✓ Disease-, drug- or radiation-induced immunosuppression

●Patients with comorbidities:

- ✓ Previous prosthetic joint infection
- ✓ Insulin-dependent (type 1) diabetes
- ✓ First 2 years after joint replacement
- ✓ Malnourishment
- ✓ Hemophilia

Box 1

**** Patients at risk for poor healing and orofacial infection due to impaired immunologic function:**

This category includes, but is <u>not limited to</u>, patients with the following medical conditions:

●HIV patients with CD4+ cell counts below 200/µL or severe neutropenia (neutrophil count < 500/µL)

●Diabetics with poor glycemic control:

- ✓ Brittle diabetes (in which control is very difficult to achieve)
- ✓ Who require a high dose of insulin (in type 1 diabetes) and have FBS > 207

●Patients undergoing irradiation of the Head and Neck (to prevent osteoradionecrosis)

●For immunocompromised patients, including:

- ✓ Neutropenic cancer patients
- ✓ Patients on chemotherapy
- ✓ Hematopoietic stem cell or solid organ transplantation on immunosuppressive therapy
- ✓ Patients with end-stage renal disease treated with hemodialysis

The Absolute neutrophil count (ANC) should be checked. If ANC is lower than 1000, the antibiotic prophylaxis should be recommended.
ANC= White Blood Cell (WBC) count X % of neutrophil

Box 2
