# Supplementary material for: Designing a customized clinical practice guideline regarding antibiotic prophylaxis for Iranian general dentists
Source: BMC Oral Health. 2019 Oct 7;19:217. doi: 10.1186/s12903-019-0905-3 (PMC6781348; doi:10.1186/s12903-019-0905-3)
Supplement: Supplementary file 2 — Additional file 2: Our recommended flowchart for antibiotic prophylaxis for Iranian general dentists based on available antibiotics in Iran Part B: patients at high risk for distant-site infection. (PDF 355 kb) [file 12903_2019_905_MOESM2_ESM.pdf]

Our recommended flowchart for antibiotic prophylaxis for Iranian general dentists based on available dose of antibiotics in Iran
Part B: patients at high risk for distant-site infection

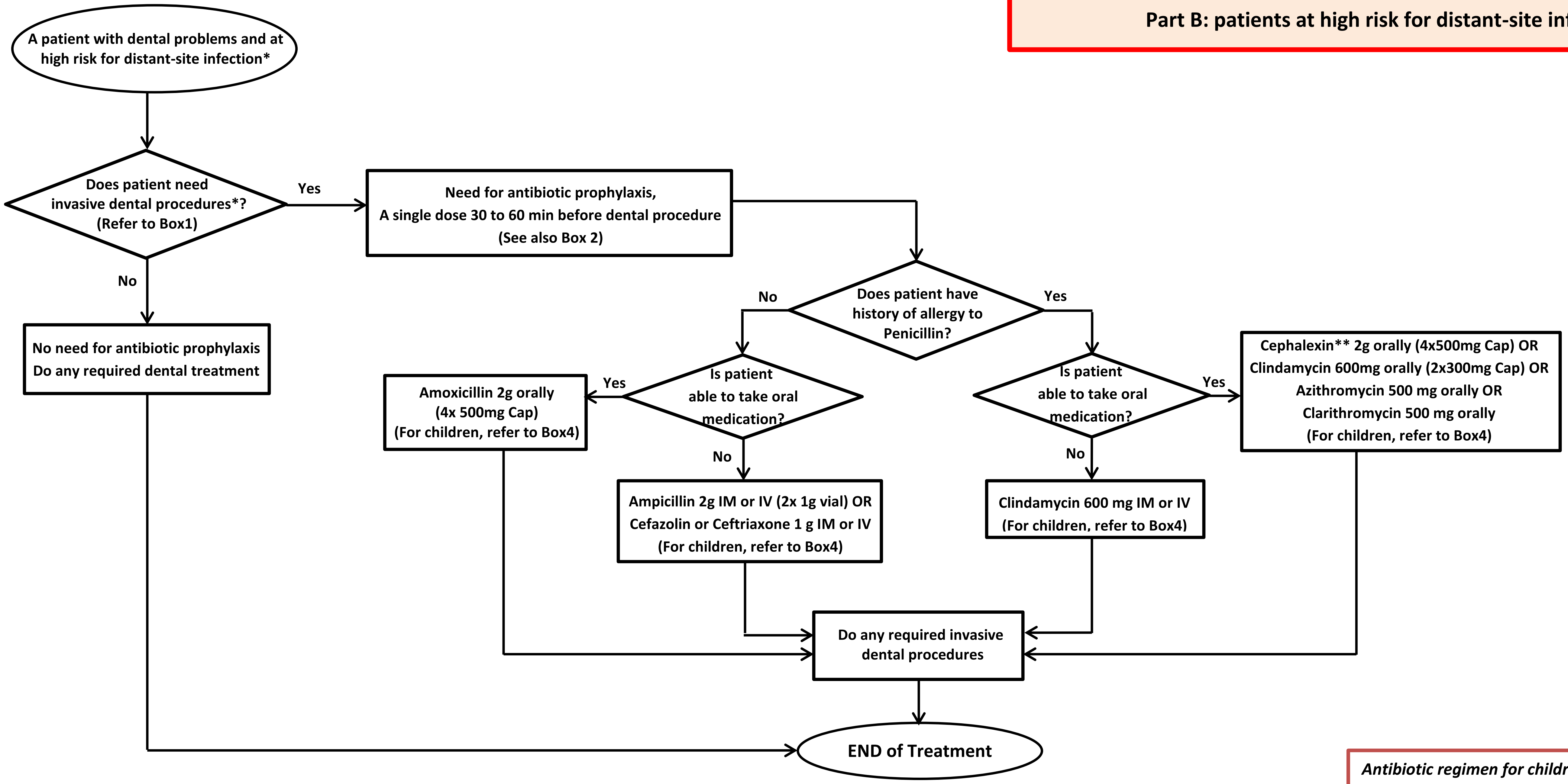

***Invasive dental procedures:**
All dental procedures that involve manipulation of the gingival tissue or the periapical region of teeth or perforation of the oral mucosa

That includes all dental procedures except the following procedures and events:
- ●Routine anesthetic injections through non-infected tissue
- ●Taking of dental radiographs
- ●Placement of removable prosthodontic or orthodontic appliances
- ●Adjustment of orthodontic appliances
- ●Shedding of deciduous teeth and bleeding from trauma to the lips or oral mucosa

Box 1

■ If the antibiotic is inadvertently not administered before the procedure, the dosage may be administered up to 2 hours after the procedure.

■ If dental procedure lasts over 6 hours; it may be prudent to administer and additional dose.

■ Allow at least 10 days between appointments (preferably 10 to 14 days). If this is not possible, select an alternative antibiotic prophylaxis regimen for appointments within a 7-day period.

Box 2

Antibiotic regimen for children:

- ●Amoxicillin: 50 mg/kg orally
- ● Ampicillin: 50 mg/kg IM or IV
- ●Azithromycin: 15 mg/kg orally
- ●Cefazolin or Ceftriaxone: 50 mg/kg IM or IV
- ●Cephalexin: 50 mg/kg orally
- ● Clindamycin: 20 mg/kg orally or IM or IV
- ● Clarithromycin: 15 mg/kg orally

**Cephalosporins should not be used in an individual (child or adult) with a history of anaphylaxis, angioedema, or urticaria with penicillins or ampicillin.

Box 3
