# Supplementary figures and images for: Designing a customized clinical practice guideline regarding antibiotic prophylaxis for Iranian general dentists
Source: BMC Oral Health. 2019 Oct 7;19:217. doi: 10.1186/s12903-019-0905-3 (PMC6781348; doi:10.1186/s12903-019-0905-3)

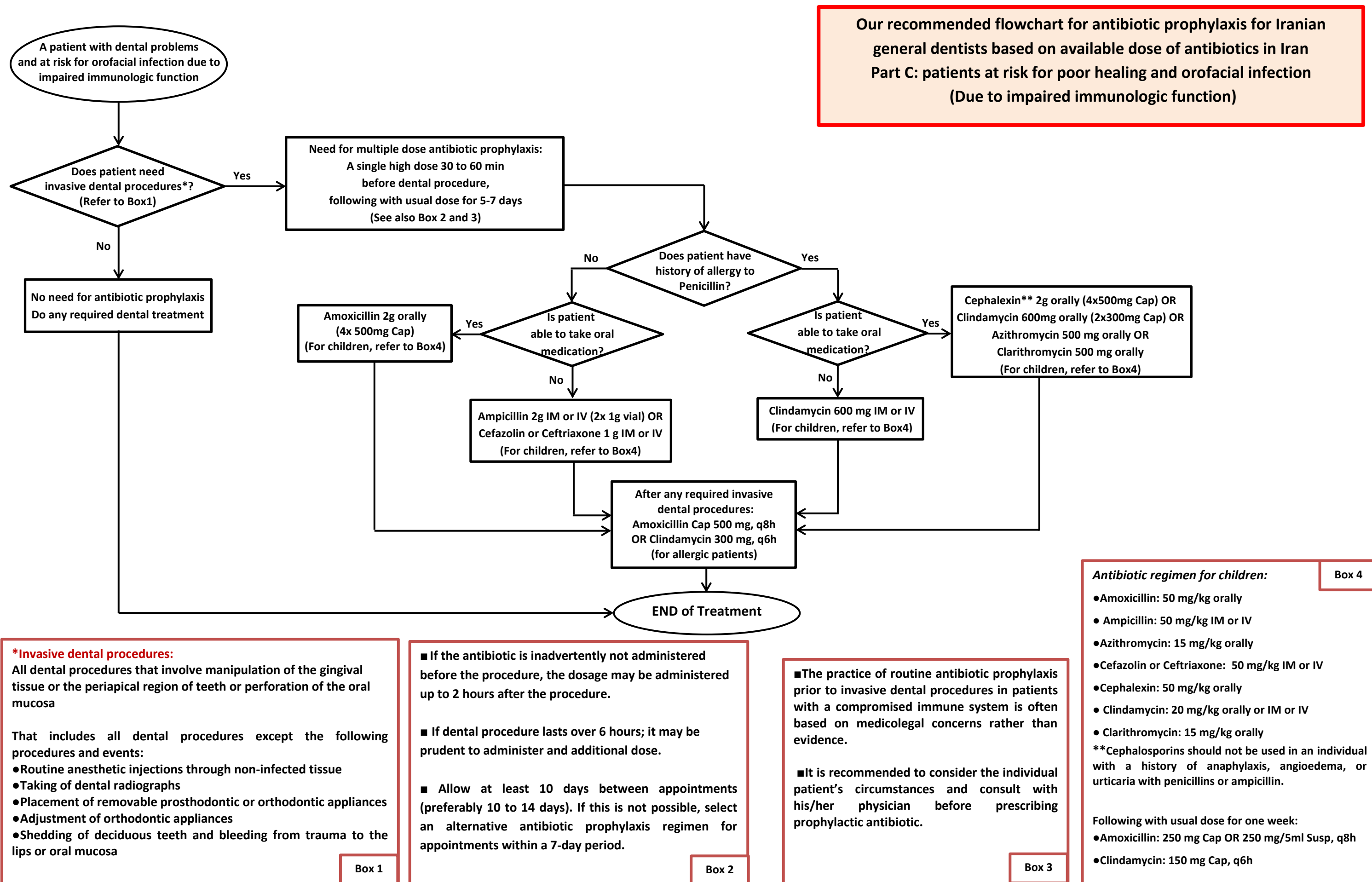

Supplement: Supplementary file 3 — Additional file 3: Our recommended flowchart for antibiotic prophylaxis for Iranian general dentists based on available antibiotics in Iran Part C: patients at risk for poor healing and orofacial infection (Due to impaired immunologic function). (PDF 357 kb) [file 12903_2019_905_MOESM3_ESM.pdf]
